# From Theory to Practice: Leveraging Chemical Principles To Improve the Performance of Peroxydisulfate-Based In Situ Chemical Oxidation of Organic Contaminants

**DOI:** 10.1021/acs.est.3c07409

**Published:** 2023-12-18

**Authors:** Lenka McGachy, David L. Sedlak

**Affiliations:** † Department of Environmental Chemistry, University of Chemistry and Technology Prague, Technická 5, 16628 Prague, Czech Republic; ‡ Department of Civil and Environmental Engineering, University of California, Berkeley, California 94720, United States

**Keywords:** in situ chemical oxidation, peroxydisulfate, PDS, ISCO, organic contaminants

## Abstract

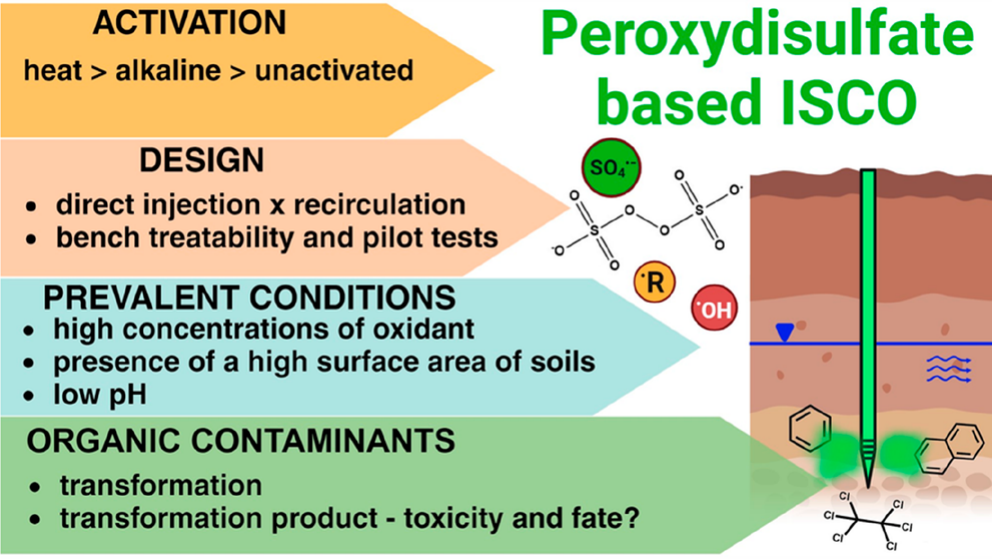

In situ chemical oxidation (ISCO) using peroxydisulfate has become more popular in the remediation of soils and shallow groundwater contaminated with organic chemicals. Researchers have studied the chemistry of peroxydisulfate and the oxidative species produced upon its decomposition (i.e., sulfate radical and hydroxyl radical) for over five decades, describing reaction kinetics, mechanisms, and product formation in great detail. However, if this information is to be useful to practitioners seeking to optimize the use of peroxydisulfate in the remediation of hazardous waste sites, the relevant conditions of high oxidant concentrations and the presence of minerals and solutes that affect radical chain reactions must be considered. The objectives of this Review are to provide insights into the chemistry of peroxydisulfate-based ISCO that can enable more efficient operation of these systems and to identify research needed to improve understanding of system performance. By gaining a deeper understanding of the underlying chemistry of these complex systems, it may be possible to improve the design and operation of peroxydisulfate-based ISCO remediation systems.

## 1 Introduction

Starting in the mid-1970s, the discovery of widespread soil and groundwater contamination led to the investment of large sums of money in site remediation. By the early 1990s, this often resulted in the removal of concentrated contaminant sources using dig-and-haul and pump-and-treatment technologies.^1^ The high cost of these remedial approaches and the desire to avoid long-term liability led to the development of active strategies, like biostimulation, permeable reactive barriers, and soil vapor extraction. Although these methods were effective, they often proved to be expensive or incapable of achieving the required cleanup goals. For this reason, more aggressive in situ remediation technologies, particularly those targeting source zones, started to be implemented in the early 2000s.^2^

Among the various new remediation approaches, in situ chemical oxidation (ISCO) became popular as a means of remediating soils and shallow aquifers contaminated with halogenated solvents,^3,4^ benzene, toluene, ethylbenzene, and xylenes (BTEX),^5,6^ petroleum hydrocarbons,^7^ polynuclear aromatic hydrocarbons (PAHs),^8,9^ pesticides,^10−12^ chlorobenzenes,^13,14^ and polychlorinated biphenyls (PCBs).^15^

ISCO typically involves the introduction of a solution containing a relatively high concentration (i.e., 0.05–1.25 M) of a strong oxidant, such as permanganate (MnO_4_^–^), hydrogen peroxide (H_2_O_2_) or a peroxydisulfate salt, into an injection well. This is often accompanied by recovery of the injection fluid at one or more recovery wells.^16−18^ Fluids extracted from recovery wells are pumped into a holding tank and amended with a concentrated stock solution of the oxidant. Sufficient residence time is provided in the aquifer to ensure that only treated water is reinjected into the aquifer.^19^

The rate at which the oxidant decomposes in the subsurface is critical to the success of ISCO because it determines the delivery of reactive species to the target zone. For example, reactions of H_2_O_2_ with soil minerals and organic matter tend to take place over a period of minutes to hours in the subsurface,^20,21^ resulting in a limited transport distance for injected oxidants under many conditions. To slow these processes, stabilizers (e.g., KH_2_PO_4_ or sodium phytate) are used to keep H_2_O_2_ from decomposing too quickly.^22,23^ In addition, under certain circumstances (e.g., high concentrations of oxidant) ISCO can enhance the transfer of contaminants from soil to groundwater by oxidizing organic coatings on mineral surfaces and increasing groundwater temperature (H_2_O_2_ decomposition in soils is an exothermic reaction).^24,25^ Furthermore, the decomposition of H_2_O_2_ can produce O_2_ bubbles (2H_2_O_2_ → 2H_2_O + O_2_) that can increase the rate of transfer of volatile contaminants from the saturated zone to the vadose zone without oxidizing them.^26^ In cases where this process releases high concentrations of contaminants, vapor recovery systems are used to minimize contaminant release.^26^ Logistical consideration associated with well construction and the transfer of contaminants to the vadose zone often limits the use of ISCO to soils and shallow groundwater where injection and recovery is possible (typically depths less than 25 m^25^).

Among the different oxidants employed for ISCO, solutions of peroxydisulfate salts (e.g., Na_2_S_2_O_8_, K_2_S_2_O_8_) became popular due to their relatively low cost, relative stability, and water solubility. After injection, peroxydisulfate anion (S_2_O_8_^2–^) undergoes reactions that result in the formation of nonselective reactive oxidant species (ROS), primarily sulfate radicals (SO_4_^•–^) that can be further converted into hydroxyl radicals (·OH). Except for fully halogenated compounds (e.g., CCl_4_, hexachlorobenzene) and a few other contaminants, SO_4_^•–^ and ·OH can oxidize most contaminants of concern.^27,28^

S_2_O_8_^2–^-based ISCO has been a topic of considerable interest among researchers.^29^ Numerous empirical studies have been conducted by remediation engineers (i.e., bench-scale treatability tests) as part of the process of determining appropriate S_2_O_8_^2–^ doses and the need for additives (e.g., pH adjustment). Academic researchers also have studied the mechanisms of SO_4_^•–^-radical-based reactions to gain insight into kinetics and transformation products. However, academic research is rarely conducted under the conditions that are relevant to ISCO (i.e., high concentrations of oxidant, presence of high concentrations of mineral surfaces, and solutes that can affect radical chain reactions). The objective of this Review is to use the results of available studies and knowledge of the actual conditions employed in ISCO to gain insight into the chemistry of S_2_O_8_^2–^-based ISCO that can be used to operate these systems more efficiently.

## 2 Peroxydisulfate Chemistry

**Figure 1:**
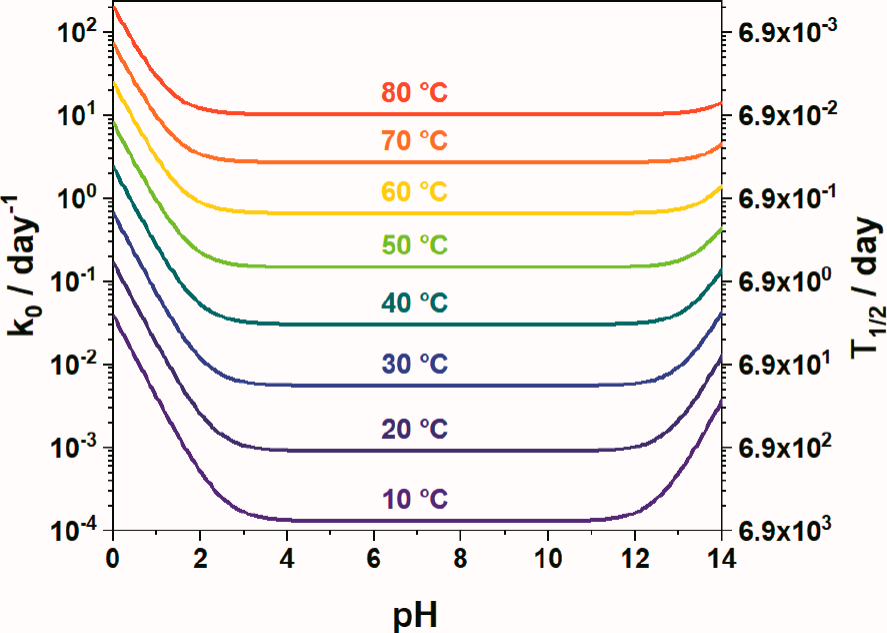
Calculated kinetics of S_2_O_8_^2–^ decomposition in aqueous solution as a function of the pH and temperature. The calculations are based on activation energies and pre-exponential factors of partial reactions (eqs 2, 3 and 8).^36^

Peroxydisulfate salts dissociate in water to form S_2_O_8_^2–^, a species that is relatively stable in aqueous solutions. In the absence of other solutes, S_2_O_8_^2–^ slowly oxidizes water through a series of radical reactions with the net stoichiometry shown in eq 1. In the absence of catalysts of other solutes, the half-life for S_2_O_8_^2–^ at circumneutral pH values is around 14 months at 25 °C. S_2_O_8_^2–^thermolysis (eq 2) exhibits a strong temperature dependence (i.e., the activation energy of the reaction is 128.9–140.2 kJ mol^–1^^30^). As a result, the rate of eq 2 can be accelerated by heating; at 50 °C the half-life of S_2_O_8_^2–^ decreases to about 5 days. In weakly basic and neutral solutions, eq 2 is the main process contributing to the initiation of S_2_O_8_^2–^ decomposition. Under acidic and strongly basic conditions, S_2_O_8_^2–^ decomposes by two simultaneous reactions, one uncatalyzed (eq 2) and the other catalyzed by hydrogen (eq 3) and hydroxide (eq 8) ion, respectively.^31,32^ However, strong acidic (pH < 3) and basic (pH > 12) conditions are required to contribute significantly to S_2_O_8_^2–^ decomposition (Figure 1).
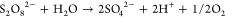
1
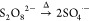
2
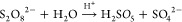
3

Under many conditions, the first step in the decomposition of S_2_O_8_^2–^ is thermolysis to produce two SO_4_^•–^ ions (eq 2). In the absence of high concentrations of contaminants, most of the SO_4_^•–^ reacts with water (i.e., eq 4), hydroxide (eq 5) or S_2_O_8_^2–^ (eq 6). The combined reactions of thermolysis (eq 2) and radical scavenging (eqs 4–6 and reactions with organic contaminants) result in low, steady-state SO_4_^•–^ and ·OH concentrations during S_2_O_8_^2–^ decomposition. The main factors responsible for the variations in SO_4_^•–^ and ·OH concentrations are usually the concentration of S_2_O_8_^2–^ and pH. Under conditions typical of ISCO, characterized by neutral or acidic pH levels, the majority of SO_4_^•–^ does not undergo conversion into ·OH,^33^ making SO_4_^•–^ the predominant radical.

Organic contaminants typically do not become dominant sinks for the radicals until concentrations become high. The rate of S_2_O_8_^2–^ decomposition may be altered by the presence of organic contaminants for several reasons. First, some compounds directly react with S_2_O_8_^2–^, usually through 2-electron oxidation reactions (eq 7) to produce sulfate (SO_4_^2–^) and an oxidized organic compound. These reactions tend to be slow at temperatures typical of ISCO systems, but for electron-rich compounds, partial oxidation can occur through direct reactions. For example, the second-order rate constants for oxidation of phenols and anilines by S_2_O_8_^2–^ at 30 °C typically range from 10^–1^ to 10^–4^ M^–1^ s^–1^.^34^ In the presence of 0.1 M S_2_O_8_^2–^ (i.e., a concentration typical of ISCO systems), the half-life of these compounds would range from approximately 1 min to 19 h. In addition, the strong temperature dependence of these reactions assures that this process is considerably faster at the higher temperatures used during heat activation of S_2_O_8_^2–^.^35^

The second reason that the rate of S_2_O_8_^2–^ decomposition often changes in the presence of organic contaminants is related to reactions of organic compounds with SO_4_^•–^ and ·OH. Second-order rate constants of SO_4_^•–^ reactions with organic contaminants range from 10^7^ to 10^10^ M^–1^ s^–1^,^37^ and ·OH reacts with organic contaminants with second-order rate constants that range from 10^8^ to 10^11^ M^–1^ s^–1^.^38^ These reactions initiate a series of radical reactions that can be more important to peroxydisulfate decomposition than the reaction pathways that dominate in the absence of solutes.
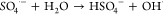
4
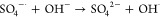
5
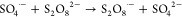
6
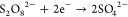
7

Although S_2_O_8_^2–^ can be converted into SO_4_^•–^ by thermolysis (eq 2), the rate of radical production is usually too slow to be useful for remediation under ambient conditions encountered in groundwater. As described in the following sections, technologies are available for heating the aquifer during ISCO. As an alternative, the rate at which S_2_O_8_^2–^ is converted into SO_4_^•–^ can be enhanced by raising the pH of the groundwater through the addition of a strong base (e.g., NaOH). The initial step of the alkaline activation process is the hydroxide-initiated fission of S_2_O_8_^2–^ to form peroxymonosulfate (SO_5_^2–^) and SO_4_^2–^ (eq 8). A similar fission of the S–O bond in SO_5_^2–^ results in the formation of a hydroperoxyl anion (HO_2_^–^) (eq 8), followed by nucleophilic attack of HO_2_^–^ on the peroxide (O–O) bond in S_2_O_8_^2–^, resulting in the formation of SO_4_^•–^ and superoxide (O_2_^•–^) radical (eq 10).^39^ Simultaneously, SO_5_^2–^ decomposes to form SO_4_^2–^ and O_2_ (eq 11).^40^
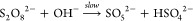
8
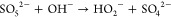
9
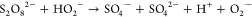
10
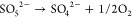
11For the alkaline hydrolysis pathway to enhance the rate of conversion of S_2_O_8_^2–^ to SO_4_^•–^ and ·OH (via eq 5) to rates that are useful for remediation, pH values greater than 12 must be achieved in the groundwater. It is challenging to maintain such high pH values in the subsurface due to the buffering by carbonates and other soil minerals, as well as the release of H^+^ as S_2_O_8_^2–^ is converted into SO_4_^2–^ by various pathways (e.g., eq 1). As a result, alkaline activation typically requires the addition of large volumes of concentrated base into the subsurface, resulting in complicated logistics and possible health hazards to workers when the injection fluids are handled.^41,42^

The addition of dissolved transition metals (e.g., Fe^2+^) has been researched as a means of converting S_2_O_8_^2–^ into SO_4_^•–^. However, the use of Fe^2+^ for S_2_O_8_^2–^ activation within ISCO appears to be impractical. Fe^2+^ reacts quickly with S_2_O_8_^2–^ through a reaction analogous to the Fenton reaction, but unlike the Fenton-like processes that enable Fe to catalyze radical formation by cycling between the +II and +III oxidation states, S_2_O_8_^2–^ and the radicals that it generates under neutral and low pH conditions do not result in a catalytic reaction. Thus, Fe^2+^ activation of S_2_O_8_^2–^ would require the addition of relatively large quantities of iron. In addition, injected Fe^2+^ is converted into Fe^3+^, which is insoluble at neutral pH.^43^ It may be possible to avoid precipitation by adding a chelated form of Fe(II) into the aquifer.^44,45^ Nevertheless, chelating agents that are commonly used to prevent Fe(III) precipitation (e.g., citric acid, EDTA) are not Fe-specific and may complex with and mobilize other metals from the soil matrix.^46^ Other methods for S_2_O_8_^2–^ activation also appear to be impractical under conditions relevant to ISCO. Carbon-based materials^47^ and copper and cobalt oxides^48^ can catalyze the conversion of S_2_O_8_^2–^ into SO_4_^•–^, but they are relatively expensive and cannot easily be adapted to deliver catalysts to porous media.^49,50^

The homolytic cleavage of the O–O bond that produces two SO_4_^•–^ (eq 2) can be accelerated by increasing the temperature in the subsurface.^51^ Although energy demanding, heat activation is considered one of the most promising methods for employing S_2_O_8_^2–^ for ISCO.^52−54^ One benefit of thermal activation is that it allows engineers to determine the location in the aquifer where S_2_O_8_^2–^ activation takes place.^3,55^ In addition, it avoids the need to handle additional chemicals (e.g., NaOH) and minimizes concerns about their impact on the subsurface environment.^55^

To thermally activate S_2_O_8_^2–^ in the field, various techniques have been employed, including in situ thermal treatment using steam, resistive heating and preceding S_2_O_8_^2–^ addition by application of H_2_O_2_, which generates heat as it is converted into O_2_ and ·OH.^18,56,57^ Although these approaches have some advantages over base addition, they face their own set of challenges. For example, it is difficult to achieve uniform steam distribution in the subsurface, and steam generation can be complicated if the water used requires the addition of softeners to prevent the buildup of calcium, lime and rust in the steam lines.^18,58^ Electrical resistance heating provides better heat distribution in the subsurface, but it requires installation of electrodes, which can greatly increase project costs.^59,60,53^ Coupling S_2_O_8_^2–^ application with H_2_O_2_^56^ addition tends to be the least expensive and most practical approach for heating the subsurface because it uses much of the same infrastructure (i.e., injection well, piping) that is employed for S_2_O_8_^2–^-based ISCO. To thermally activate S_2_O_8_^2–^, H_2_O_2_ is injected before S_2_O_8_^2–^, and the recommended dosage is 1 mol of H_2_O_2_ per 1–2 mol of S_2_O_8_^2–^ (the higher the H_2_O_2_ dosage, the shorter is the S_2_O_8_^2–^ lifetime).^61^

The critical parameter of a thermally activated PDS-based ISCO is temperature. Most laboratory studies of S_2_O_8_^2–^ use for oxidation of organic contaminants are conducted at temperatures of 30–90 °C.^62−66^ Higher temperatures result in rapid production of ROS as well as fast rates of contaminant oxidation. However, in the context of ISCO applications, lower temperatures (30–40 °C) may be more suitable because heating an aquifer to temperatures above 40 °C is expensive and rapid activation also limits the distance that S_2_O_8_^2–^ can travel.^52−54^ As shown in Figure 1, the lifetime of peroxydisulfate is relatively short at elevated temperatures and can be even shorter in the presence of soil, which limits the persistence of PDS in the subsurface and thus the distance over which thermally activated PDS can be effective. Moreover, reducing the temperature of treatment decreases energy consumption.

## 3 Design of S_2_O_8_^2–^-Based ISCO Remediation Systems

Key factors determining the cost, complexity, and logistics of operating S_2_O_8_^2–^-based ISCO systems include oxidant volume and initial S_2_O_8_^2–^ concentration, well spacing, depth and location, injection rates, and the duration, timing, and number of injection events. The design of ISCO systems is determined by the nature of the contamination, cleanup goals and uncertainties in subsurface conditions, including soil heterogeneity and properties affecting mass transfer rates.^25^ The collection of extensive data on subsurface conditions can reduce some of these uncertainties. Before implementing S_2_O_8_^2–^-based ISCO, it is important to assess the potential for treatment to affect downgradient water sources (e.g., by mobilizing toxic metals or other contaminants). This typically involves land assessment and bench treatability or pilot-scale treatability tests. These tests can offer insights into the risks of contaminant mobilization for the specific site, as well as design information regarding the required amount of S_2_O_8_^2–^ and the rate at which the oxidant will be consumed in the subsurface.

**Figure 2:**
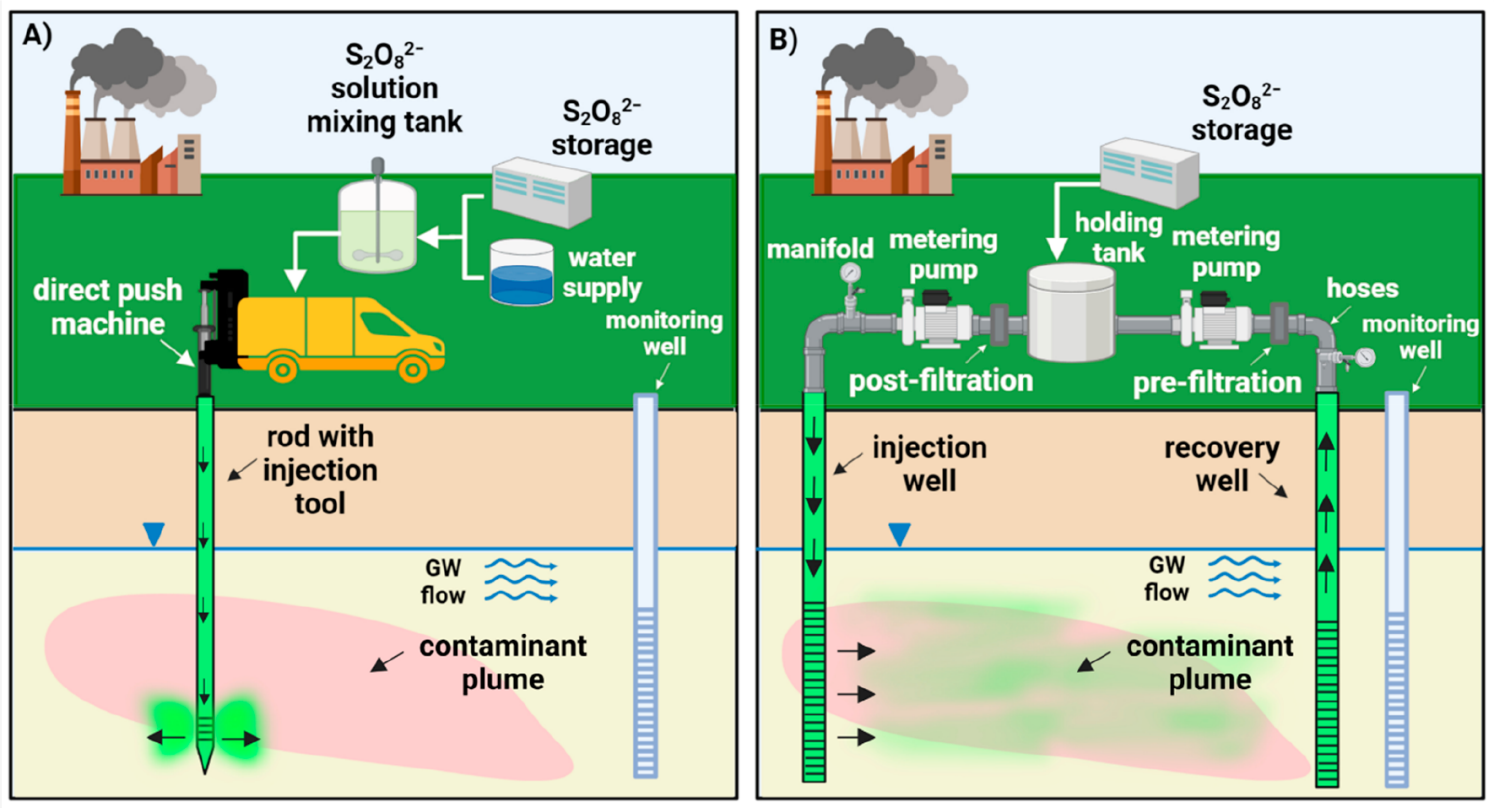
Conceptual illustration of (A) direct injection process and (B) recirculation system.

The success of many ISCO projects depends on the ability of the injection system to distribute the oxidant into the subsurface in a uniform manner. Two approaches are typically used to deliver the S_2_O_8_^2–^ solution into the subsurface: direct injection (Figure 2A) and recirculation (Figure 2B).^57^ Direct injection involves mixing of solid S_2_O_8_^2–^ with a specified volume of water from an external source above ground followed by injection into the aquifer without any effort to recover or recirculate the oxidant (Figure 2A). Because S_2_O_8_^2–^ is injected at high concentrations, the possibility of acidity, metals mobility, and S_2_O_8_^2–^ and/or SO_4_^2–^ migration to nearby receptors should be assessed. Nonetheless, it is well documented that biotic and abiotic attenuation mechanisms reduce the impact of these processes to levels considered safe by regulatory agencies in a period of 1 to 4 months.^18,26^

The direct injection process is mostly performed with temporary push points (Figure 2A); however, permanent injection wells can also be used. The typical direct push method involves using a machine with a hydraulic hammer that pushes small-diameter metal rods with an injection tool to the targeted treatment depth. This process can be performed using either a top-down and/or bottom-up injection sequence. As direct push technologies are mounted on a platform, the access of large or small vehicles to the injection area and the injection depth (typical maximum depth is 25 m) must be considered.

For recirculation systems, an S_2_O_8_^2–^-containing salt is dissolved in groundwater extracted from the contaminated zone. After mixing in a holding tank which also serves as an above-ground treatment unit, the treated groundwater is reinjected. The recirculation systems can be designed in several different configurations. Typically, as shown in Figure 2B, injection wells are installed in the upgradient portion of the plume, and recovery wells are located downgradient of the plume. This strategy is particularly useful for treatment of contaminants that are not strongly sorbed to soil and aquifer materials. The mixing that occurs between S_2_O_8_^2–^ and the groundwater in the above-ground treatment unit ensures that the contaminants are exposed to oxidants. In addition, recirculation enhances the distribution of an oxidant in the subsurface, provides hydraulic control in the treatment zone (e.g., develop a concentration gradient, containment), and ensures that any excess oxidants or products of oxidation reactions (e.g., acid, SO_4_^2–^, metals, and oxidation byproducts) are captured. Above-ground infrastructure consists mainly of metering pumps, prefiltration elements to remove sediments from extracted groundwater, a holding tank equipped with a mixer, a post-filtration element to remove solids from the injected fluid, system manifolds to distribute the flow and allow measurement and adjustment of injection/extraction pressures and flow rates, and hoses connecting the facility to the injection and recovery wells. When volatile contaminants (e.g., vinyl chloride) are treated, the tank is typically equipped with a blower to withdraw air from the headspace of the tank and discharge it through the activated carbon unit into the atmosphere. In general, water is reinjected without further additional treatment (e.g., acid neutralization), but in some situations (e.g., injection upgradient of a drinking water aquifer), additional treatment may be employed.

Although recirculation systems have numerous advantages over direct injection systems, the process often consumes more oxidant. It is also more complicated to operate and requires expensive above-ground infrastructure (e.g., hosing, tanks, electricity, and filtering of solids), making it less cost-effective just for small sites.^16^ Typically, higher volumes (e.g., 40–85% of a pore volume of the entire treatment area) and lower concentrations (0.05–0.2 M) of S_2_O_8_^2–^ are used when injecting via recirculation, whereas lower volumes (e.g., ∼1% of a pore volume of the entire treatment area) and higher S_2_O_8_^2–^ concentrations (0.5–1.25 M) are applied when using direct injection.^18^ The number of pore volumes that will be injected or recirculated depends on site-specific factors such as oxidant dispersion/diffusion.^57^

Another factor affecting the cost and design of S_2_O_8_^2–^-based ISCO systems is the duration of deployment. The direct injection typically takes place over a period of 4 h to 2 days with S_2_O_8_^2–^ lasting in the ground for between 30 and 90 days, depending upon the volume and injection rates, while recirculation is typically completed over a period of 4–8 weeks.^18,67^ Due to the difficulty associated with achieving contact between S_2_O_8_^2–^ and contaminants adsorbed onto clay or organic-rich particles, multiple injection events (redosing) are often necessary to achieve cleanup goals. The number of redosing events is typically determined by data collected after the injection event.^25^ ISCO is considered one of the most rapid in situ remediation technologies, with times required to remediate a site on average 1–3 years.^68^ In general, remediation of simple sites (e.g., sandy aquifers, small sites without strongly adsorbed contaminants) is easier compared to sites with low permeability formations or sites with extensive contamination.^67^

Although solid S_2_O_8_^2–^ is a very stable material, excessive heat or moisture can result in decomposition of the stored material (e.g., material at the bottom of a pallet or in the center of a drum). As heat generated may stimulate self-accelerating decomposition, S_2_O_8_^2–^ should be stored in a cool, clean, dry place away from point sources of heat (e.g., radiant heaters) and moisture (e.g., rain). When working with caustic chemicals (e.g., NaOH) as an activator, it is imperative to apply increased precautionary measures. Additionally, materials such as soft metals (e.g., copper) can be degraded by concentrated S_2_O_8_^2–^ solutions.^57^ Therefore, the materials used for the delivery system should be compatible with long-term PDS exposure (e.g., stainless steel, high-density polyethylene, and polyvinyl chloride). Similarly, at sites where large volumes of S_2_O_8_^2–^ are injected, daylighting (surfacing of groundwater) can occur. Thus, a spill containment plan also should be developed as part of the remedial action work plan.^69^

The cost of S_2_O_8_^2–^-based ISCO is highly site-specific, as it is influenced by the various parameters mentioned above. The total cost of remediation can be divided into two components. The first component is the capital cost, which is a fixed project expense and includes costs associated with site preparation (e.g., utility lines and concrete pad installation), mobilization/demobilization (including transportation to the site, storage, fabrication, assembly, setup, and dismantling), equipment acquisition, and labor (for transportation and setup on site if not already included in mobilization). The second component is operating cost, which is variable and includes mainly expenditure on equipment purchase/rental (e.g., storage tanks and pumps), chemicals/reagents, energy, waste disposal and labor required to operate the system. In general, capital costs increase with site size and range from approximately $170,000 for smaller sites (with a target aquifer volume of approximately 900 m^3^) to approximately $1,300,000 for larger sites (with a target aquifer volume of approximately 34,700 m^3^).^18^ Overall, these costs are competitive with alternative remediation techniques and offer the benefit of being completed more quickly (i.e., successful ISCO projects can be completed in several months, whereas pump-and-treat and bioremediation often require several years to decades to complete.

## 4 Challenges Affecting the Performance of S_2_O_8_^2–^-Based ISCO Systems

Although laboratory studies show that S_2_O_8_^2–^ can be used to oxidize contaminants under well-controlled conditions, the effectiveness of this technology under field conditions is often limited by several practical issues. By understanding the practical limitations of using S_2_O_8_^2–^-based ISCO, it may be possible for researchers to develop more effective ways to predict when this approach will work, increase its effectiveness, and develop approaches to minimize the unintended consequences of its use.

### 4.1 Delivering S_2_O_8_^2–^ to Low Permeability Zones

Irrespective of the oxidant employed, the success of all ISCOs often depends upon subsurface geology. Due to the heterogeneity in the subsurface and the presence of layers or lenses of low-permeable minerals (e.g., clay), dead-end pores or stagnant zones,^70^ oxidants may not reach contaminated zones that require treatment. Under these conditions, oxidants will preferentially flow through areas of least resistance (e.g., highly permeable sand layers), resulting in incomplete oxidation of contaminants.^71^ This is especially problematic for hydrophobic contaminants that tend to associate with particulate organic matter and clays,^72^ especially under conditions in which contaminants have been present for several decades.^73^ To ensure that oxidants have the opportunity to come into contact with contaminants in these difficult-to-reach zones, efforts are needed to maximize the longevity of the oxidant in the subsurface.^27,74^

Failure of ISCO systems to deliver oxidants to contaminated zones often results in the rebound effect, as contaminants gradually diffuse out of clay- and organic-matter-rich zones after oxidant application ends. In response, researchers have developed techniques to deliver S_2_O_8_^2–^-containing fluids to low-permeability zones using electrokinetic techniques.^55,54,75,76^ Although these techniques have the potential to help with the delivery of S_2_O_8_^2–^ to low-permeability zones, their widespread application in these approaches has not made substantial progress.

### 4.2 Loss of S_2_O_8_^2–^ through Reactions with Aquifer Solids

Researchers who have studied the fate of H_2_O_2_ in ISCO systems have found that transition metal oxides and soil organic matter can cause rapid loss of oxidant.^77,78^ Consequently, they hypothesized that the same phenomenon could lead to the loss of S_2_O_8_^2–^ in ISCO systems. However, laboratory studies and field observations indicate that S_2_O_8_^2–^ is much less susceptible to loss by reactions with aquifer solids. Nevertheless, there are situations in which oxidant loss occurs through reactions with geologic materials.

**Figure 3:**
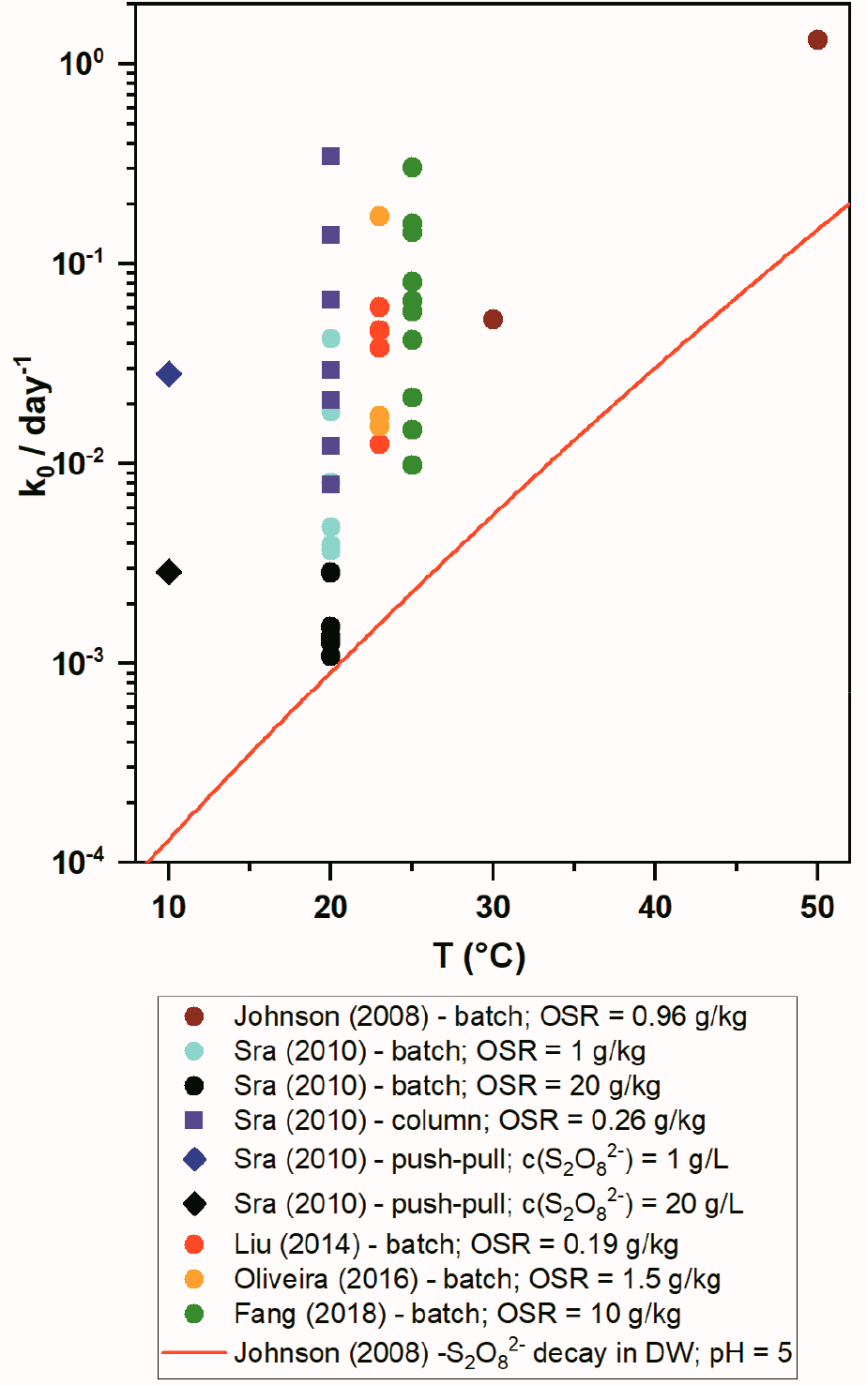
Observed first-order kinetic rate constants of S_2_O_8_^2–^ decomposition in different soils and minerals^27^ under different thermal conditions and experimental designs (batch, column, push–pull) compared with calculated kinetics of S_2_O_8_^2–^ decay in aqueous solution at pH 5^36^.^27,36,79−,81^ OSR is oxidant-to-soil mass ratio.

S_2_O_8_^2–^ is very stable in pure sand, having a half-life of more than 1 year.^27,81^ However, as shown in Figure 3, the rate of S_2_O_8_^2–^ decomposition accelerates in the presence of more reactive minerals and surfaces. Half-lives of S_2_O_8_^2–^ decrease to less than a year when Fe(III) and Mn(IV) oxides comprise more than about 1% of the aquifer solids.^27,80−82^ In addition, the loss of S_2_O_8_^2–^ increases significantly when the oxidant comes into contact with pure minerals (e.g., ferrihydrite, pyrolusite).^27^ Metal oxides rarely account for more than 10% mass of aquifer solids on a mass basis. Therefore, the rates of reactivity of S_2_O_8_^2–^ with pure minerals exaggerate the importance of this loss mechanism.

Similarly, if the organic matter in the soil is more than about 1% of the mass of aquifer solids, the decomposition rate of S_2_O_8_^2–^ also increases (Figure 3), with half-lives dropping to less than ∼30 days.^81^ However, the fraction of soil organic matter in aquifer solids tends to be much less than 1%, typically <0.01%.^83−85^ Under these conditions, the loss of S_2_O_8_^2–^ by direct reactions with organic matter is likely negligible.

In addition, increasing the S_2_O_8_^2–^ concentration from low concentrations typically used in laboratory experiments (e.g., 0.05 M^81^) to the high concentrations employed in the field (e.g., 0.2 M) results in an increase of half-life by more than an order of magnitude^79^ (Figure 3, push–pull).

### 4.3 Biogeochemical Impacts and Mobilization of Metals of S_2_O_8_^2–^ Injection

Application of S_2_O_8_^2–^-based ISCO alters the geochemistry of the subsurface, mainly due to the production of acid and the release of sulfate.^32^ Although large quantities of acid are generated as the injection fluid decomposes, the pH of groundwater after treatment typically ranges from 2 to 5 due to the neutralization of protons by minerals (e.g., CaCO_3(s)_) associated with the aquifer material.^18,26^ Furthermore, the neutralization of acidity is not instantaneous; pH values typically increase over several days after treatment ends. In well-buffered systems, pH values eventually return to preinjection pH values. However, at sites with a paucity of minerals capable of neutralizing persistent acidity, pH values below 2 have been observed.^18^

The drop in pH often results in the release of toxic trace elements and/or the formation of new mineral species. Elevated concentrations of trace elements such as arsenic, chromium and vanadium have been reported in treatment zone wells where S_2_O_8_^2–^-ISCO was employed even after pH values returned to preinjection levels.^18^ This has been a concern with respect to chromium because the relatively nontoxic Cr(III) can be converted into its carcinogenic form, Cr(VI), during the oxidation process.^86^

Acid produced during the decomposition of S_2_O_8_^2–^ can also lead to the formation of minerals that alter the permeability of the subsurface. For example, Ca(II) released when CaCO_3(s)_ dissolves under acidic conditions can react with SO_4_^2–^ to produce gypsum (CaSO_4_·2H_2_O), which is relatively insoluble.^87^ Under acidic conditions, Fe(III) and Mn(IV) oxides also will dissolve. Because these minerals often play a role in maintaining soil structure and can precipitate when the acidity is neutralized, the permeability of the subsurface can change after S_2_O_8_^2–^-ISCO.

In addition to producing acidic conditions, the high concentrations of SO_4_^2–^ and other dissolved ions (e.g., Na^+^, Ca^2+^) produced during the decomposition of S_2_O_8_^2–^ and subsequent acid-catalyzed dissolution processes can degrade water quality. S_2_O_8_^2–^-based injection fluids typically contain over 1000 mg/L of SO_4_^2–^, which is considerably higher than the secondary drinking water standard for SO_4_^2–^ of 250 mg/L. In addition, under anaerobic conditions SO_4_^2–^ can be reduced to sulfide (HS^–^), compromise water quality and result in the precipitation of sulfide minerals.

Changes in pH and redox potential induced by the injection of S_2_O_8_^2–^-based fluids can also affect microbial communities. Microorganisms that are sensitive to oxidative treatments, including anaerobic organisms such as organohalide-respiring bacteria (e.g., *Dehalococcoides mccartyi*, *Dehalobacter*, *Geobacter*, and *Desulfitobacterium*), will experience a decrease in abundance and activity.^88^ In contrast, the population and activity of microbes that are less sensitive to the alteration in redox potential (e.g., facultative anaerobes) might remain unchanged or even exhibit positive responses.^89−91^ For example, it has been demonstrated that acidification (pH < 3) and an increase in redox potential (>500 mV) associated with S_2_O_8_^2–^ treatment disrupt the microbial community, with full recovery of anaerobic bacteria such as *Dehalococcoides mccartyi* taking longer than half a year.^90^ The recovery is likely due to the recolonization of the previously oxidized area by organisms moving in groundwater from the upgradient direction or the reintroduction of microorganisms into a treated area, potentially originating from blind pockets or low-permeability zones.^92,93^ Additionally, the observed increase in biomass following the application of S_2_O_8_^2–^ oxidant could be linked to the increased presence of bioavailable oxidation byproducts.^94^

### 4.4 Effect of Sorption on Contaminant Transformation Rates during S_2_O_8_^2–^-Based ISCO

Observations based upon laboratory studies and field applications indicate that S_2_O_8_^2–^-based ISCO is less effective in the treatment of organic contaminants that are strongly sorbed to solids.^95,96^ The inefficiency of the oxidation processes is related to the fact that most SO_4_^•–^ or OH· are produced in solution (e.g., when thermal activation is employed) or at the mineral–water interfaces (e.g., when S_2_O_8_^2–^ is activated by processes taking place on mineral surfaces). Particle-associated organic contaminants tend to exhibit low reactivity with aqueous SO_4_^•–^ or OH· radicals because the oxidants are consumed by other dissolved species (i.e., natural organic matter, HCO_3_^–^, and S_2_O_8_^2–^) before they can diffuse to the interfaces where particle-associated organic contaminants are located.^97^ In addition, uncharged organic contaminants often partition into particulate organic matter and organic coatings on minerals; this polymeric organic material would compete with contaminants for reactive oxidant species like SO_4_^•–^. Thus, rates of transformation of absorbed contaminants will often be limited by the rates at which they are released to solution to reestablish equilibrium partitioning after dissolved forms of the compounds are depleted.

The rate of desorption of neutral organic contaminants from the solid phase depends upon the hydrophobicity of the compound as well as the amount of organic carbon and surface area of the material from which the solute is associated.^98,99^ Although elevated temperatures encountered during heat-activated SO_4_^•–^ treatment result in increased concentrations of hydrophobic compounds in the aqueous phase and faster desorption kinetics, this effect tends to be relatively unimportant. For example, an increase of temperature from 25 to 50 °C will only increase the equilibrium concentration of dissolved pyrene by about 45% assuming an enthalpy of sorption (Δ*H*_sorp_) of −11.8 kJ/mol.^100^

To enhance the efficacy of ISCO in the treatment of hydrophobic organic compounds, researchers have employed surfactants.^101−103^ However, this approach has not been widely adopted in practice due to a variety of factors. First, most surfactants react with SO_4_^•–^ or OH·; competition for radicals can slow the rate of contaminants oxidation. In addition, there is evidence that some surfactants (e.g., Brij 35, Triton X-100, and Tween 80) slow the rate of S_2_O_8_^2–^ activation by interrupting radical chain reactions that are important to the oxidation process.^104−106^ Finally, residual surfactants remaining after treatment can compromise the water quality.

### 4.5 Effect of Dissolved Solutes on the Kinetics of S_2_O_8_^2–^-Based ISCO

Much of the power of ISCO treatment with oxidants can be traced to the occurrence of radical chain reactions, which can be divided into three phases. After the initial production of primary radicals (e.g., SO_4_^•–^), a radical propagation phase is initiated as stable molecules (e.g., organic contaminants and inorganic solutes) are converted into radicals. These secondary radicals (e.g., OH·, carbon-centered radicals) eventually react with S_2_O_8_^2–^ to propagate the radical chain or react with other radicals to form a stable nonradical adduct and terminate the chain reactions.

The kinetics of S_2_O_8_^2–^ activation as well as the efficiency through which contaminants are transformed are affected by dissolved solutes, such as chloride (Cl^–^), bicarbonate (HCO_3_^–^), and natural organic matter. The way in which these species affect the efficacy of the treatment processes depends upon their concentrations, which, in turn, is often determined by local conditions (e.g., pH, mineral types, and presence of cocontaminants).

Cl^–^ reacts with SO_4_^•–^ quickly (Table 1, eq 12) to form chlorine radical (Cl·) as the primary product, which in turn initiates a chain of propagation reactions (e.g., eqs 13 and eq 14 in Table 1).^107,108^ However, the rate constant for the reverse of eq 13 is also fast, which can push the reaction backward. Therefore, there is usually little loss of SO_4_^•–^ through this process unless chloride concentrations are very high.^109,110^ If Cl^–^ are present at an increased concentration compared to the initial S_2_O_8_^2–^ (e.g., [Cl^–^] > 0.3 M, [S_2_O_8_^2–^] > 0.023 M), the forward reaction (eq 13) would be significant and would lead to a greater level of sulfate radical scavenging.^111^ Generally, Cl^–^ concentration in groundwater is less than 0.015 M,^112^ and S_2_O_8_^2–^ is used at substantially higher concentrations during ISCO. Therefore, Cl^–^ usually will not be a significant sink for SO_4_^•–^ during S_2_O_8_^2–^-based ISCO treatment.

**Table 1:**
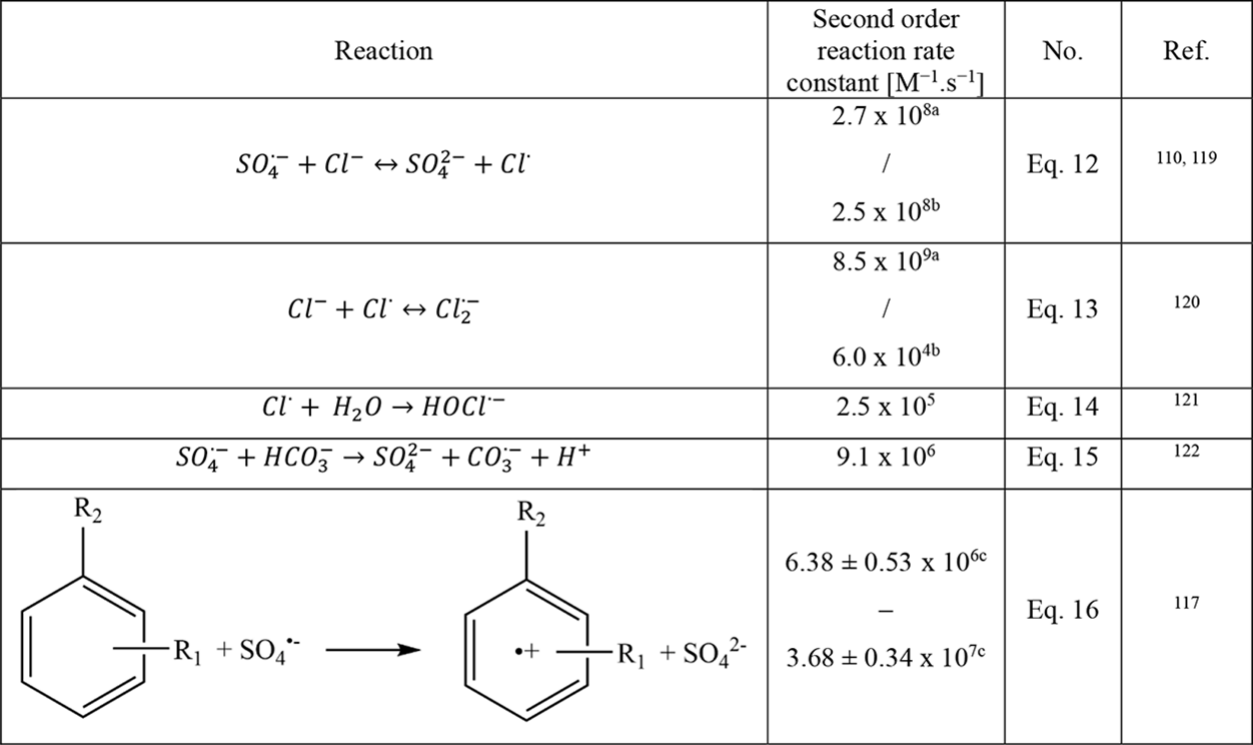
Important Reactions Involving SO_4_^•–^ and Dissolved Species Typically Encountered during ISCO

a Forward.

b Backward.

c M_C_^–1^ s^–1^.

Similarly, HCO_3_^–^ also can scavenge SO_4_^•–^ (e.g., eq 15 in Table 1) and impact reaction pathways, kinetics, and the overall efficiency of oxidative treatment.^113,114^ The concentrations of HCO_3_^–^ in groundwater depend on the nature of minerals through which the groundwater flows (e.g., typically around 1 mM in granitic rock or sandy aquifers and ∼10 mM in limestone formations).^115^ Under the low pH conditions typical of S_2_O_8_^2–^-based ISCO (i.e., <5), H_2_CO_3_* will be the predominant form of dissolved inorganic carbon.^116^ Thus, the scavenging of SO_4_^•–^ by carbonate species will be negligible. If base activation is used for S_2_O_8_^2–^ activation, scavenging could be more significant.

In addition, dissolved organic matter presented in groundwater can react with SO_4_^•–^ (eq 16 in Table 1). If dissolved organic matter is presented at high concentrations compared to the initial molarity of S_2_O_8_^2–^ (e.g., 25 mg_C_/L, 0.005 M S_2_O_8_^2–^), its scavenging of SO_4_^•–^ can become important.^117^ However, typical dissolved organic matter concentrations in groundwater (i.e., 1–2 mg_C_/L^118^ are considerably lower than the amount of S_2_O_8_^2–^ employed in ISCO.

## 5 Transformation of Organic Contaminants

There are three main mechanisms through which organic contaminants are transformed during S_2_O_8_^2–^-based ISCO: (1) direct reaction with S_2_O_8_^2–^ (i.e., two-electron transfer), (2) reactions with SO_4_^•–^, and (3) reactions with secondary radicals.

### 5.1 Two-Electron Transfer Mechanism

Due to its strong electrophilic character, S_2_O_8_^2–^ is capable of oxidizing electron-rich moiety organic contaminants (e.g., substituted phenols, arylamines) without the occurrence of radical chain reactions (Elbs and Boyland–Sims oxidation).^123,124^ The two-electron transport reaction usually occurs via two steps: (i) attack of S_2_O_8_^2–^ at the carbon atom located ortho/para to the electron-donating moiety, accompanied by deprotonation, resulting in formation of phenolate/arylamine sulfate ions, followed by (ii) hydrolysis to form hydroxylated aromatics and sulfate.^34^ Based on the Behrman’s mechanism, S_2_O_8_^2–^ reacts with phenols and arylamines in two ways depending on whether or not the compound is protonated.^125^ Under alkaline conditions (e.g., pH ≈ 13), *o*-phenolsulfates/*o*-aminoarylsulfates are formed, while under acidic conditions phenols and amines polymerize to form polyphenols/polyanilines. Recent studies, however, suggest the formation of polyarylamine precipitates as well as soluble *o*-aminoarylsulfates over a broader pH range (i.e., >5).^126,127^

### 5.2 Transformation of Organic Contaminants by SO_4_^•–^ and Secondary Radicals

**Figure 4:**
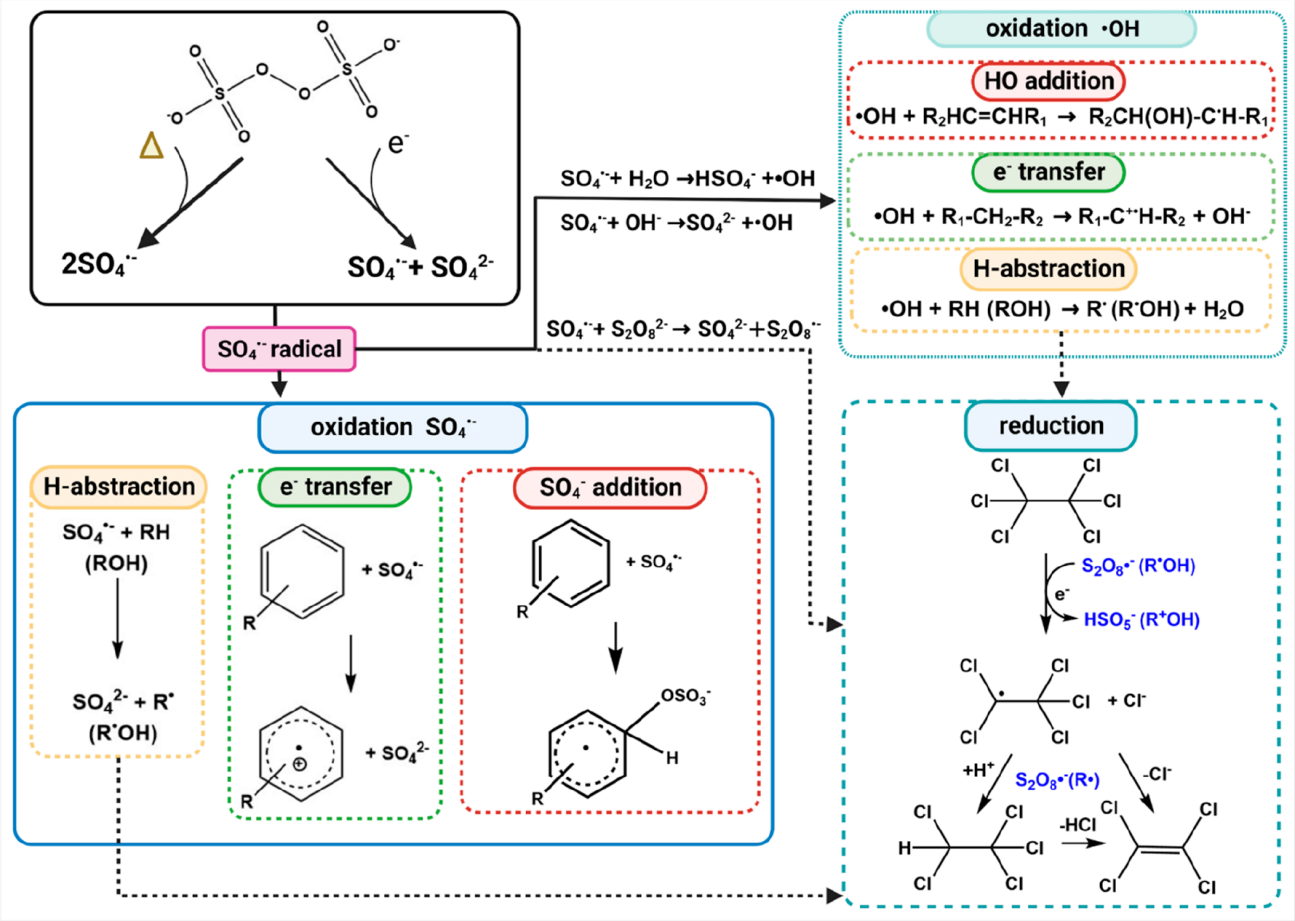
Oxidation of organic contaminants by SO_4_^•–^ and product radicals. The process is initiated by homolytic (Δ) or heterolytic (e^–^) cleavage of the O–O bond in S_2_O_8_^2–^ and generation of SO_4_^•–^. Subsequently, SO_4_^•–^ reacts with organic compounds to produce secondary organic radicals. SO_4_^•–^ can react with water or hydroxide to generate ·OH, which can oxidize many contaminants of concern. Due to eq 5, the conversion of SO_4_^•–^ into ·OH is especially significant at high pH values (e.g., during base activation). However, under neutral or acidic pH, which are typical of ISCO, most of SO_4_^•–^ reacts with other solutes before it can be converted to ·OH. Additionally, the reaction of SO_4_^•–^ with S_2_O_8_^2–^ produces S_2_O_8_^•–^ that together with other radicals such as α-hydroxyalkyl radicals can in the absence of O_2_ reduce highly chlorinated compounds. Created with BioRender.com.

SO_4_^•–^ radicals react with organic molecules and solutes by three mechanisms:^128^ (i) hydrogen atom abstraction, (ii) single-electron transfer, and (iii) addition to double or triple bonds, leading to the formation of secondary radicals that undergo a suite of reactions leading to a variety of different products (Figure 4). Furthermore, these reactions may occur simultaneously.

For example, SO_4_^•–^ reacts with alkylated benzenes (i.e., BTEX) by single-electron transfer from the attacked species to SO_4_^•–^ radical, resulting in the formation of the benzene radical cations and SO_4_^2–^.^129−131^ The next step involves the addition of OH^–^ to the benzene-derivative radical cation that then forms a hydroxycyclohexadienyl radical.^132^ This radical then can react with O_2_ to produce hydroxycyclohexadienyl peroxy radicals, which then can eliminate hydroperoxyl radical (HO_2_^•^) to produce hydroxylated products (eq 17).^133^ Alternatively, hydrogen abstraction can produce hydroquinone radical and then hydroquinone (eq 18).^134,135^ In an alternative pathway, the hydroxycyclohexadienyl peroxyl radical can also undergo oxygen addition to a double bond to form a bicyclic peroxy radical, which in the absence of any oxidant may be disproportionated to produce two endoperoxides. Both products are unstable and can undergo ring opening and production of 5- and 6-membered unsaturated aldehydes and ketones (eq 17),^136,137^ which can be further oxidized to carboxylic acid and finally CO_2_. Similar reactions can also be triggered by the reaction of aromatic compounds with ·OH.^138−140^ In addition, SO_4_^•–^ also reacts with aromatic compounds through radical addition (Figure 4). Although the addition process is usually considered a secondary pathway,^141,142^ it leads to the formation of organosulfates, which may be of environmental concern due to their higher mobility in the subsurface compared to the hydrophobic parent compounds as well as their elevated toxicity.^143,144^ However, organosulfates may undergo further transformation, such as further reaction with SO_4_^•–^ and ·OH or biotransformation, resulting in complete mineralization (i.e., conversion to CO_2_).^137,145^
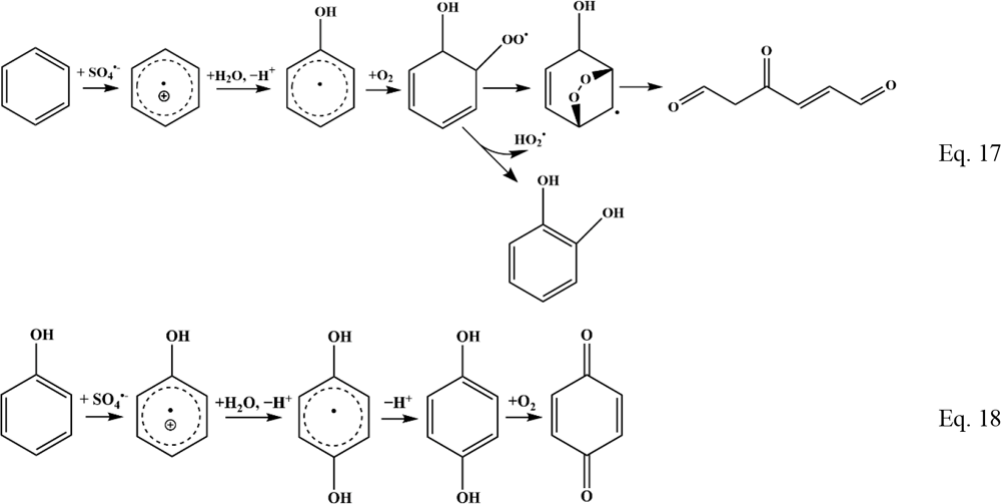
17

Similarly, polyaromatic hydrocarbons react with SO_4_^•–^ predominantly by single-electron transfer, leading to the formation of oxygen transformation products (i.e., aromatic ketones, aromatic quinones, and aliphatic or aromatic acids), which can further react with SO_4_^•–^ and ·OH^8^ or undergo biotransformation.^146^

Alternatively, hydrogen abstraction of a carbon-bond hydrogen atom is believed to be the main mechanism by which SO_4_^•–^ reacts with tertiary dialkyl ethers (methyl and ethyl *tert*-butyl ether, and methyl *tert*-amyl ether) to generate carbon centered radicals, which then react with O_2_, forming peroxyl radicals that undergo an acid-catalyzed hydrolysis reaction to yield tertiary alcohols and carboxylic acids.^65^

Similarly, haloalkanes (e.g., CH_2_Cl–CHCl_2_) react with SO_4_^•–^ via abstraction of a carbon-bond hydrogen atom, resulting in the formation of alkyl-like radicals (e.g., CH_3_–C^•^Cl_2_).^114^ These radicals react with O_2_ to produce peroxyl radicals (e.g., CH_3_–C(Cl_2_)OO^•^) that may hydrolyze to corresponding carboxylic acid, HCl, and superoxide radical (O_2_^•–^).^147^

S_2_O_8_^2–^-based ISCO is not a very effective means of treating highly halogenated organic contaminants (e.g., polychlorinated benzenes, polyhalogenated C_1_ and C_2_ compounds) under oxic condition.^10,64,148^ This is related to the low reactivity of carbon–halogen bonds with electrophilic radicals (e.g., SO_4_^•–^ and ·OH).^149,150^ However, highly halogenated contaminants more readily undergo reductive dehalogenation reactions, creating the possibility that reducing radicals produced during the chain decomposition of S_2_O_8_^2–^ could produce transformation products that react with SO_4_^•–^ and ·OH.

SO_4_^•–^ reacts with S_2_O_8_^2–^ to produce a persulfate radical (S_2_O_8_^•–^) (eq 6). In the absence of O_2_, S_2_O_8_^•–^ reacts with S_2_O_8_^2–^, initiates radical chain reactions (eq 19) and reduces highly chlorinated contaminants like hexachloroethane^151^ and DDT.^12^ In addition, the consumption of one molecule of S_2_O_8_^2–^ by S_2_O_8_^•–^ produces one molecule of SO_4_^•–^, which again can react with S_2_O_8_^2–^ to produce S_2_O_8_^•–^. However, in the presence of O_2_, the reaction between S_2_O_8_^•–^ and O_2_ terminates the reaction (4S_2_O_8_^•^ + O_2_ + 2H_2_O → 8HSO_5_^–^ + 4H^+^). Another approach involves the use of carbon-centered radicals that are produced when SO_4_^•–^ and •OH radicals react with short chain length aliphatic compounds (e.g., methanol, ethanol). It has been reported that these radicals can reduce highly halogenated compounds such as carbon tetrachloride or hexachloroethane.^151,152^ However, O_2_ competes with contaminant carbon-centered radicals (e.g., ·CH(CH_3_)OH + O_2_ → CH_3_CHO + HO_2_^•^).^153^ HO_2_^•^ and its conjugate base, O_2_^•–^, then quickly undergo bimolecular dismutation to produce H_2_O_2_ under acidic conditions.^154−156^ Since S_2_O_8_^2–^ decomposition produces O_2_ (eq 1), it is necessary to use sufficient quantities of alcohol to ensure simultaneous reduction of halogenated contaminants and initiate chain decomposition of S_2_O_8_^2–^ that produces additional carbon-centered radicals (eq 20) and consumes O_2_. In this first phase the reactions of carbon-centered radicals result in the formation of partially dehalogenated products that contain electron-rich double bonds or carbon–hydrogen bonds (Figure 4) and are less susceptible to further reduction but are prone to subsequent oxidation by SO_4_^•–^ and ·OH.^151,157^ The following oxidation reactions require sufficient concentrations of S_2_O_8_^2–^ as well as a means of accelerating the rate of S_2_O_8_^2–^ activation.^152^

Additionally, it has been reported that perfluoroalkyl acids which are resistant to oxidation by ·OH treatment react with SO_4_^•–^ to form shorter-chain perfluoroalkyl carboxylic acids, which may be further degraded and eventually mineralized.^66,158^ The multistep mechanism responsible for the transformation of perfluorooctanoic acid to perfluoroheptanoic acid can be described by eq 21. The subsequent oxidation of perfluoroheptanoic acid to produce perfluorohexanoic acid follows an analogous mechanism. The transformation of perfluorooctanoic acid to shorter-chain perfluorocarboxylic acids by SO_4_^•–^ can only be carried out under strongly acidic conditions (pH ≤ 3).^159^
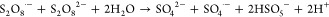
19
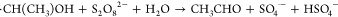
20
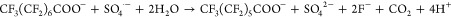
21

### 5.3 Transformation Products

The oxidation of organic contaminants by a high concentration of SO_4_^•–^ and ·OH often results in mineralization (i.e., contaminants are converted into water, carbon dioxide, Cl^–^ and other relatively innocuous products). However, the incomplete oxidation of contaminants could lead to the formation of transformation products that are more toxic and/or mobile than the compounds targeted for remediation, especially if those compounds are less reactive with SO_4_^•–^ and ·OH than the parent compounds.^160^ For example, reactions of SO_4_^•–^ and ·OH with benzene and alkylbenzenes produce toxic ring-cleavage products such as α,β-unsaturated aldehydes or ketones (e.g., acrolein).^161,137^ Additionally, incomplete transformation of haloalkanes can produce harmful haloalkenes, such as dichloromethane,^162^ which is a possible human carcinogen.^163^ Similarly, if S_2_O_8_^2–^ treatment of perfluoroalkylcarboxylic acids does not lead to complete mineralization, the resulting shorter-chain perfluoroalkylcarboxylic acids may be more mobile than the parent PFAS compounds^164^ and more difficult to remove by sorption technologies.^165^

Additionally, the oxidation of halides (X^–^) in the soil or groundwater by SO_4_^•–^ can initiate a cascade of radical chain reactions (e.g., eqs 12–14), leading to the production of secondary radicals (X^•^, X_2_^•–^, and XHO^•–^) and free halogens (X_2_/HXO).^166^ These species are extremely reactive electrophiles that can halogenate electron-rich substances, such as NOM^167^ and benzene derivates.^168^ This process may lead to the production of halogenated byproducts, such as trihalomethanes and haloacetic acids,^169−171^ which raises concerns due to their well documented human health effects.^172−175^ Field measurements made during ISCO treatment have revealed elevated concentrations of chloromethane and methylene chloride, which are precursors to trihalomethanes; however, chloroform or other trihalomethanes have not been detected at elevated concentrations after the application of S_2_O_8_^2–^.^18^ Therefore, more research is necessary to assess the formation of halogenated byproducts under the conditions typical for S_2_O_8_^2–^-based ISCO. Another concern is the potential production of chlorate (ClO_3_^–^); due to sequential oxidation of Cl^–^, concentrations that could pose a health hazard have been reported under conditions similar to those employed for ISCO.^108,176^

SO_4_^•–^ also reacts with ammonia (NH_4_^+^) and nitrate (NO_2_^–^) to produce nitrogen dioxide (NO_2_^•^) and monoxide (NO^•^) radicals, which can react with electron-rich compounds such as PAHs or NOM to produce toxic nitro-byproducts.^177−181^ For example, nitro-PAHs are known to possess higher mutagenicity and carcinogenicity compared to their parent PAHs.^182,183^ While PAHs need initial enzymatic activation before they can act as mutagens or carcinogens,^184^ their nitro-byproducts are direct acting mutagens.^185^ Similarly, nitro-aromatics pose potential hazards to ecosystems and human health, including the risk of cancer.^186^ In addition, the introduction of a nitro group into the molecule could enhance the mobility of the compound by reducing its tendency to partition onto surfaces (i.e., aquifer solids).

SO_4_^•–^ also has the potential to form organosulfonates through addition reactions. These compounds as well exhibit greater mobility in the subsurface and higher toxicity than their parent compounds.^144^ For example, *p*-cresol sulfate, a relatively polar transformation product of benzene treatment by SO_4_^•–^, is associated with renal toxicity and kidney damage.^187^

Most of the transformation products underwent further oxidative treatment with SO_4_^•–^. However, they can also undergo biodegradation. For example, nitro-aromatics can be completely mineralized via catabolic processes without the need for additional external substrates in an oxic environment.^188^ The primary mechanism of aerobic degradation of nitroaromatics is initiated by the addition of a molecular oxygen atom to the benzene ring catalyzed by oxygenase, with subsequent release of a nitro group and ring cleavage, as in the biodegradation of 4-nitrophenol.^189^ Desulfurization of aromatic sulfonates proceeds by a similar pathway.^190^ However, evidence for the biodegradation of nitro-PAHs is lacking. So far, only a few microorganisms have been identified that are able to degrade nitro-PAHs, such as nitroanthracene.^191^ Nitro-PAHs are also more resistant to further oxidation by SO_4_^•–^ than their parent compounds.^180^ Therefore, additional research is needed to better identify the formation of toxic transformation products and develop appropriate treatment methods.

## 6 Implications and Prospects

As the use of S_2_O_8_^2–^-based ISCO continues to advance, it is becoming a well-established and extensively studied field of research. This progress is evident with the increasing number of academic studies and the implementation of full-scale applications in soil and groundwater remediation. However, there are still challenges that limit the performance of this technology in field applications.

S_2_O_8_^2–^ is a relatively stable oxidant; however, there is a trade-off associated with this intrinsic stability due to its slow rate of reaction with most contaminants. Therefore, S_2_O_8_^2–^ needs to be activated to generate SO_4_^•–^ in order to oxidize most recalcitrant contaminants. Among various activation methods, thermal activation appears to be the most viable option, but the optimal temperature range (30–40 °C) must balance energy consumption and reaction efficiency. H_2_O_2_ represents a suitable method to thermally activate S_2_O_8_^2–^. However, because of its very high soil oxidant demand, using H_2_O_2_ may not be feasible for sites with a high clay content and low permeability. Heat can be introduced into the subsurface also by steam injection or with electrodes; however, these approaches are suitable mainly for smaller sites (as the size increases, so does the cost). Nevertheless, challenges still exist with heat activation, such as achieving uniform heat distribution. Hence, further research and engineering improvements are crucial to maximize the effectiveness of the heat-activated S_2_O_8_^2–^-based ISCO.

In cases where thermal activation is not feasible, alkaline activation can serve as an alternative approach. This method involves the injection of a strong base (e.g., NaOH) that raises the pH to above 12 and activates S_2_O_8_^2–^. However, there are several important considerations associated with this approach. First, careful attention must be paid to the use and safe handling of strong bases throughout the design and implementation of the project. Another critical consideration relates to pH adjustment, which consists of two key components. The first component pertains to the amount needed to adjust the pH of the soil and groundwater to the desired level. The second component involves the quantity required to counteract the impact of the acid formed during S_2_O_8_^2–^ decomposition. Therefore, both of these components must be carefully considered when determining the amount of pH modifier.

Although other methods, like employing heterogeneous catalyst or dissolved transition metals, are actively being researched, their applicability to ISCO remains limited. Solid phase catalysts can activate S_2_O_8_^2–^,^192,193^ but their use during in situ treatment is restricted by the challenges associated with delivering them to the source zone and concerns about their toxicity. Research in this area is limited, with most studies being conducted as batch, column, or box experiments.^194−196^ There is also a lack of information regarding pilot- or full-scale implementation. Concerning dissolved transition metals, only Fe(II) is a viable option. However, research^27^ suggests that this process is essentially one-way, meaning that Fe(III) does not get reduced back to Fe(II) to create a catalytic loop. Therefore, the introduction of Fe(II) into the aquifer, followed by the addition of S_2_O_8_^2–^, results in a burst of SO_4_^•–^ production, but a certain portion is consumed in oxidizing Fe(II), making it an impractical approach. It might be possible to prevent Fe(III) precipitation by introducing a chelated form of Fe(II) into the aquifer. However, common chelating agents used to prevent Fe(III) precipitation, such as citric acid and EDTA, are not specific to iron and may form complexes with other metals and mobilize them from the soil matrix.

In practice, the S_2_O_8_^2–^-based ISCO for contaminant remediation faces several limitations under field conditions. The heterogeneity of subsurface geology and the presence of low-permeability zones can hinder the delivery of S_2_O_8_^2–^ to contaminated areas requiring treatment. To increase the potential for treating difficult-to-reach low-permeability zones, methods may be needed to enhance the lifetime of the oxidant as it diffuses into these regions. Additionally, the sorption of contaminants to organic matter can limit the transformation rates during S_2_O_8_^2–^-based ISCO, especially for strongly sorbed organic contaminants.

Furthermore, potential loss of S_2_O_8_^2–^ through reactions with aquifer solids, such as Fe(III)- and Mn(IV) oxides, as well as the presence of high concentrations of organic matter and dissolved solutes, such as Cl^–^, O_2_ or organic cocontaminants, can impact the kinetics and efficiency of S_2_O_8_^2–^-based oxidation reactions. However, the use of a high concentration of S_2_O_8_^2–^ during the field application can effectively counteract these inhibitory effects.

Acidic conditions and exposure of minerals and solutes to large amounts of oxidants can alter the geochemistry of the subsurface, leading to changes in the solubility of certain inorganic species and the mobilization of metals. Furthermore, the S_2_O_8_^2–^-based ISCO can partly or fully inhibit certain microbial species, especially those that are sensitive to oxygen and acidity. Since natural attenuation often is expected to play an important role in the breakdown of residual contaminants at sites where S_2_O_8_^2–^-based ISCO is planned, permanent inhibition in microbial activity is undesirable. Therefore, treatability tests should be conducted on bench- and pilot-scale to assess the potential impact of S_2_O_8_^2–^ on geochemistry and microbial activity.

SO_4_^•–^ reacts with organic molecules through various mechanisms, leading to the formation of secondary radicals and a wide range of transformation products. Despite the success of S_2_O_8_^2–^-based ISCO in the treatment of various contaminants, the technology is not very effective when highly halogenated contaminants are present. One promising approach is to employ reductive radicals, such as carbon-centered radicals produced during chain decomposition of S_2_O_8_^2–^. However, further research is necessary to assess the impact of field application conditions (e.g., presence of aquifer solids).

Incomplete oxidation of contaminants can result in the formation of potentially more toxic or mobile transformation byproducts. Similarly, reactions with solutes may lead to the formation of products such as ClO_3_^–^ or chlorinated and nitro-containing byproducts that may pose health risks to consumers of groundwater. Furthermore, SO_4_^•–^ has the potential to form organosulfates through radical addition that may also be more toxic and mobile than the parent compounds. These byproducts may undergo substantial oxidation or be transformed by microorganisms. However, certain byproducts, such as nitro-PAHS, are resistant also to biodegradation. The formation of these difficult to treat byproducts should be carefully assessed prior to S_2_O_8_^2–^-based ISCO application.

Despite all these challenges, S_2_O_8_^2–^-based ISCO is a promising technology that can be very useful in the treatment of contaminated subsurface. We believe that understanding the practical limitations of S_2_O_8_^2–^-based ISCO reviewed in this paper can guide researchers and remediation engineers in developing more effective approaches, predicting its applicability, enhancing its effectiveness, and minimizing unintended consequences when it is applied in the field.
